# Orbito-Cranial Trigeminal Schwannoma: A Case Report

**DOI:** 10.7759/cureus.86346

**Published:** 2025-06-19

**Authors:** Mario Benvenutti-Regato, Misael Salazar-Alejo, Jose Antonio Figueroa Sanchez, Pablo J Avalos-Montes, Hector R Martinez, Caroline Guerrero-de-Ferran

**Affiliations:** 1 Escuela de Medicina y Ciencias de la Salud, Tecnologico de Monterrey, Monterrey, MEX; 2 Instituto de Neurología y Neurocirugía, Centro Médico Zambrano-Hellion, San Pedro Garza García, MEX

**Keywords:** neurilemmoma, ophthalmic schwannoma, orbital schwannoma, orbital tumor, trigeminal schwannoma

## Abstract

The authors present a case of an orbito-cranial trigeminal schwannoma treated with a right orbitozygomatic approach, resulting in near-total tumor resection. This case highlights the utility of the orbitozygomatic approach, the challenges associated with achieving complete resection, and the need for careful surgical planning due to the tumor's complex anatomy. Additionally, we discuss prognosis, barriers to total resection, and existing evidence gaps. Given the rarity of orbito-cranial schwannomas, multicenter collaborations are critical to address gaps in surgical strategies, the role of radiotherapy and endoscopic approaches, and long-term outcomes.

## Introduction

Trigeminal schwannomas (TS) are rare, benign, slow-growing tumors arising from the Gasserian ganglion, the trigeminal roots, or one of the three peripheral branches of the trigeminal nerve (CN-V) [1,2]. These tumors account for 0.8-8% of all schwannomas and approximately 1% of orbital tumors [1,3,4]. The clinical presentation of TS varies depending on tumor size and location, with the hallmark involvement of the CN-V. Patients typically present with trigeminal nerve dysfunction, manifesting as pain, paresthesia, and numbness, with headache and diplopia following in frequency [1,4-7].

Several classification systems have been developed to categorize TS and guide treatment strategies. The first classification, introduced by Jefferson et al. in 1955, categorized TS based on their anatomical origin [8]. With advances in imaging, Samii et al. proposed a new classification system in 1995, focusing on the tumor’s location relative to the middle and posterior cranial fossae [1]. Four years later, Yoshida and Kawase published a classification system emphasizing the anatomical extension of the tumors [2]. The last classification system, published in 2008 by Ramina et al., modified Samii et al.'s classification to further highlight the tumor’s origin and its patterns of extension [4]. Orbito-cranial schwannomas are classified as type D (Samii et al.), type E (Yoshida and Kawase), and type IV (Ramina et al.), each representing the highest category within each system due to their extracranial extension. This underscores the complexity of these tumors and the need for a combined orbito-cranial surgical approach.

The preferred treatment is complete or near-total tumor resection, achieved in over 70% of cases due to advancements in skull-based surgical techniques and microsurgical dissection [4,9]. Surgical approaches vary based on tumor location and classification, with options including retrosigmoid, orbitozygomatic, frontotemporal, and, more recently, endoscopic endonasal techniques [10,11]. Evidence regarding the optimal management of orbito-cranial schwannomas is limited, and the outcomes of patients with incomplete resections remain unclear [12].

## Case presentation

A 36-year-old female with a six-month history of painless right ptosis presented to our institution for neurosurgical evaluation. Physical examination revealed right eye (OD) proptosis and impaired abduction, accompanied by marked binocular diplopia in the nasal and superior visual fields. Visual acuity was 20/20 in both eyes (OU), with isochoric and normoreactive pupils. Color vision, assessed with Ishihara plates, was preserved. The anterior segment examination was unremarkable, but fundoscopy demonstrated subtle blurring of the optic disc margins in the OD.

**Figure 1:**
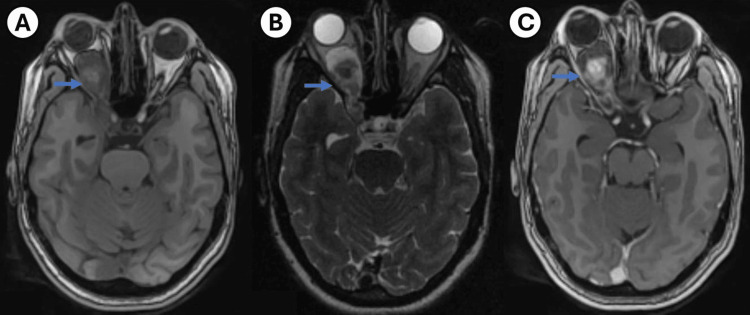
Brain MRI images Panels A and B show significant proptosis of the OD, with noticeable pupil deviation on the same side. The signal intensity within the intraconal space is highlighted, revealing its connection to the intracranial area through the optic foramen (blue arrows). Panel C displays heterogeneous contrast uptake in the inner portion of the intraconal lesion (blue arrow).

Brain MRI revealed a dumbbell-shaped extra-axial tumor measuring 32 mm x 28 mm x 8 mm. The intraconal portion exerted significant mass effect on the optic nerve, causing the observed atrophy, while the intracranial portion extended through the superior orbital fissure into the cavernous sinus (CS) (Figure 1).

A multidisciplinary team, comprising experts from neurosurgery, neuro-ophthalmology, oculoplastics, and neuroradiology, convened to determine the optimal treatment strategy. A right orbito-zygomatic craniotomy was selected due to the tumor's multicompartmental extension, particularly its infiltration of the CS. This approach offers several advantages, including a wide operating field for CS access and minimal brain retraction. These factors contribute to a higher rate of complete tumor removal with reduced morbidity, all within a single operation [5,13].

**Figure 2:**
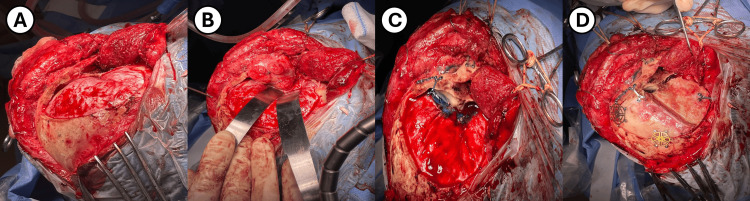
Intraoperative images A. Fronto-temporal craniotomy was performed prior to completing the orbitozygomatic resection. B. Exposure of the intraconal lesion after removing the roof and lateral wall of the orbit, as well as the zygoma. C. Reconstruction of the orbitozygomatic portion of the craniotomy using fibrin glue at the base. D. The image illustrates a temporal muscle flap used for the reconstruction.

An interfascial dissection through the skin, subcutaneous tissue, fascia, and muscles overlying the right fronto-temporo-zygomatic area was performed. Subsequently, the dissection of the periorbita was performed while preserving the orbital fat. A fronto-temporo-sphenoidal craniotomy was performed, extending medially toward the supraorbital notch. To optimize visualization and surgical reach, an orbitozygomatic extension was performed by removing the roof and lateral wall of the orbit, along with the zygoma using three osteotomies (Figure 2).

An intracranial portion of the tumor and its relationship with critical neurovascular structures in the CS were exposed (Figure 2). Microdissection techniques were employed to navigate around these critical areas and achieve a near-total (90%) resection of the tumor. However, during the resection of the intracavernous portion, significant bleeding was encountered, which compromised visualization and increased the risk of damaging nearby structures, thus preventing gross total resection (GTR) of the tumor. Periorbital reconstruction was performed using a collagen dura substitute, and the bone flaps were repositioned (Figure 2). Postoperatively, visual acuity remained unchanged. However, the patient developed paralysis of the trochlear nerve (CN-IV) and abducens nerve (CN-VI), along with partial oculomotor nerve (CN-III) palsy; findings likely attributable to intraoperative manipulation of the cranial nerves. Additionally, the patient developed a right-sided House-Brackmann (H-B) grade IV facial paralysis, possibly resulting from nerve retraction during the orbitozygomatic approach.

**Figure 3:**
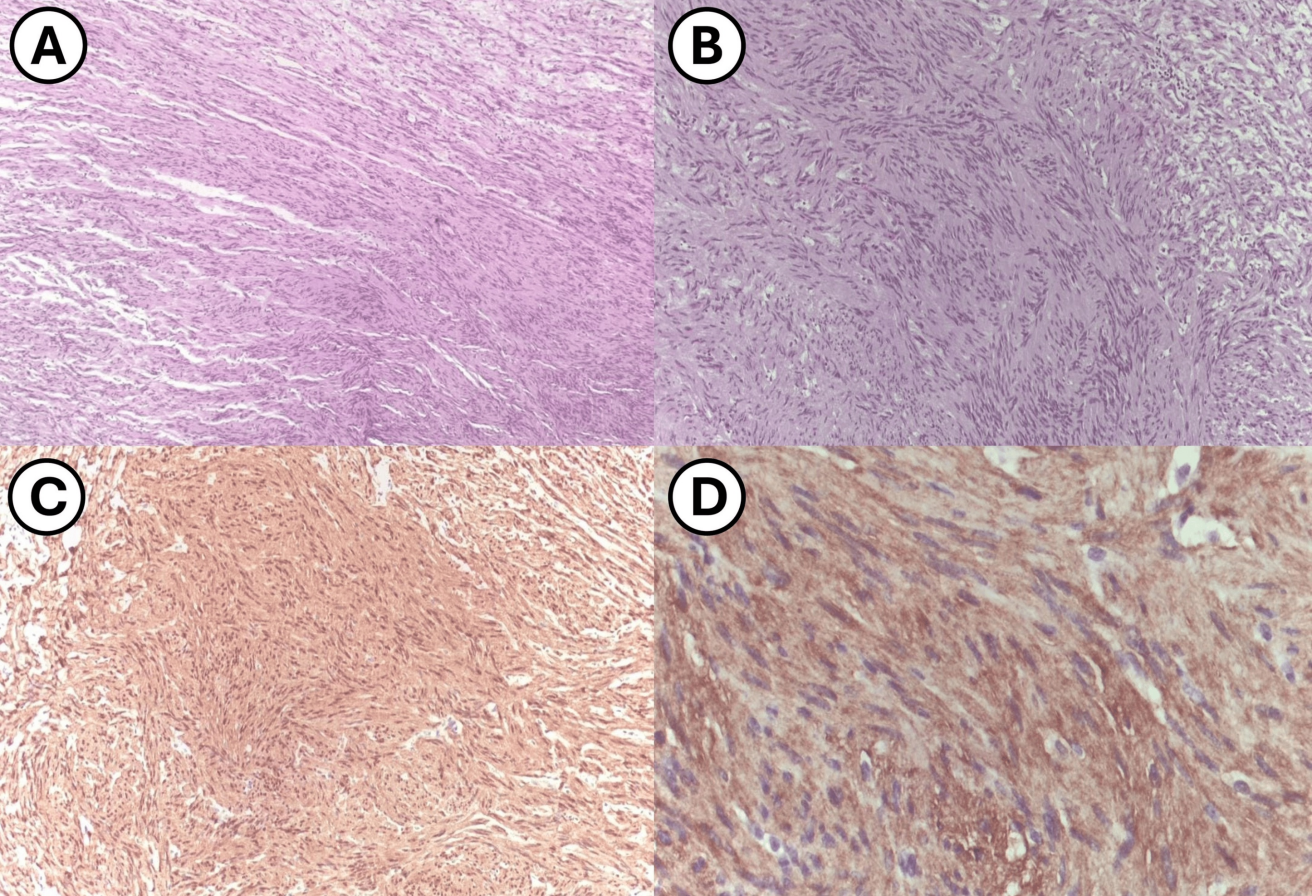
Immunohistochemistry study results A. Compact proliferation of spindle-shaped cells with wavy nuclei, showing no signs of atypia or necrosis (H&E, 100X). B. In some areas of the tumor, cells form palisades or whorls, with nuclei aligned perpendicularly (H&E, 100X). C. Strong positivity of cells for the S-100 protein (IHC). D. Cells show strong positivity for the monoclonal marker CD56 (IHC). H&E, Hematoxilin and eosin; IHC, immunohistochemistry.

Histopathological analysis of the tumor revealed a proliferation of spindle cells with elongated nuclei, displaying the characteristic biphasic pattern of Antoni A and Antoni B areas (Figure 3). Immunohistochemical staining was positive for both S-100 and CD56 (Figure 3), with a Ki-67 index of less than 1%, consistent with a diagnosis of schwannoma of the ophthalmic branch of the trigeminal nerve.

**Figure 4:**
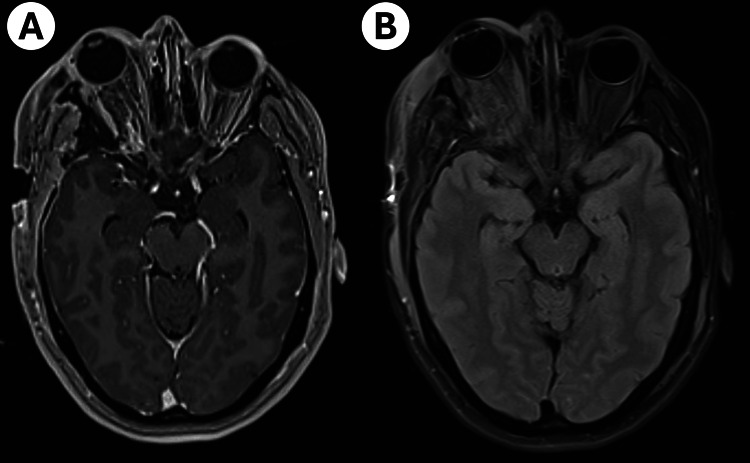
MRI images at 12-month follow-up A. Postoperative T1 with Gadolinium contrast demonstrates removal of the intraconal and intracranial components with persistent contrast enhancement. The optic nerve remains intact throughout its trajectory. B. T2 TIRM sequence reveals edema affecting the intraorbital muscles. TIRM, Turbo Inversion Recovery Magnitude.

At the 12-month follow-up, MRI demonstrated a GTR of the intraconal and extracavernous intracranial components (Figure 4). A residual intracavernous component measuring 1.5x1.3x1.3 remained, with no evidence of disease progression. The patient is currently undergoing rehabilitation and has fully recovered from the initial proptosis. Facial nerve function has significantly improved, with a current H-B score of 1, indicating near-complete recovery of CN-VII. Pupillary responses are now normal, reflecting improved CN-III function. However, CN-VI paresis persists, resulting in diplopia on extreme lateral gaze.

## Discussion

Orbito-cranial TS are exceedingly rare tumors. Two recent multicenter studies, involving eight institutions over a combined period of 40 years, documented only 12 cases. Among these, just two were managed using the orbito-zygomatic surgical approach [11,12]. Furthermore, limited information is available regarding complications, surgical outcomes, and follow-up status, particularly in cases where GTR was not achieved. Given the gaps in knowledge and the challenges to acquire high-quality evidence, a multidisciplinary approach is often needed to determine optimal management strategies on a case-by-case basis [12].

The orbito-zygomatic approach, by extending the frontotemporal osteotomy with an orbitozygomatic osteotomy, increases the angle of approach and provides a vast working space for accessing lesions involving CS, upper clivus, and adjacent vascular structures [14,15]. This technique entails the removal of the temporal bone, the zygomatic arch, and lowering of the temporal muscle, which reduces the distance to medially located lesions and allows for access in a below-upwards direction, rather than horizontally. Furthermore, removing the superior orbital rim enables access to skull base lesions from a lower point, reducing the need for frontal lobe retraction. Given the multicompartmental nature and surgical complexity of orbito-cranial schwannomas, the orbito-zygomatic approach can provide a multidirectional view, increase access, and improve the likelihood of GTR.

While outcomes after orbito-cranial schwannoma excision are generally favorable, surgery is not without risk. Common complications include reduced visual acuity, restricted eye movement, diplopia, ptosis, mydriasis, paresthesia, and facial hypesthesia [9,12]. However, these results have improved significantly in recent decades, owing to advances in microsurgery and skull-base techniques [4,10].

Despite the considerable armamentarium of surgical strategies and their continuous improvement, GTR is not possible in a considerable number of cases. Barriers to complete resection include CS involvement and attachment or close proximity to neurovascular structures, which would considerably risk their damage [1,4,5,10,13]. However, even with incomplete resection, long-term progress-free intervals are often achieved [1,11,13]. Particularly for these cases or for patients not suitable for re-resection, stereotactic radiotherapy has been investigated as a potential adjuvant therapy, but additional research is required to clearly define its role in the overall treatment strategy for these tumors [9-12]. Currently, the available evidence is insufficient to establish a standardized algorithm for selecting the optimal surgical approach. The resection strategy should be determined by considering both the neurosurgeon’s expertise and the anatomical characteristics of the orbito-cranial tumor [11].

## Conclusions

Orbito-cranial schwannomas are uncommon and complex tumors that present significant challenges in achieving GTR while minimizing postoperative neurological deficits. Key research gaps remain, including the need for a therapeutic decision-making algorithm, a better understanding of long-term prognosis, and clarity on the role of radiotherapy and endoscopic surgery. Given the rarity and heterogeneity of these tumors, multicenter collaborations are essential to gather more comprehensive and high-quality data. Such efforts will help address current evidence gaps and ultimately improve patient outcomes.
